# Enhancement of Chemical Stability and Dermal Delivery of *Cordyceps militaris* Extracts by Nanoemulsion

**DOI:** 10.3390/nano10081565

**Published:** 2020-08-09

**Authors:** Pachabadee Marsup, Kankanit Yeerong, Waranya Neimkhum, Jakkapan Sirithunyalug, Songyot Anuchapreeda, Chaiwat To-anun, Wantida Chaiyana

**Affiliations:** 1 Master’s Degree Program in Cosmetic Science, Faculty of Pharmacy, Chiang Mai University, Chiang Mai 50200, Thailand; pch.marsup@gmail.com; 2 Department of Pharmaceutical Sciences, Faculty of Pharmacy, Chiang Mai University, Chiang Mai 50200, Thailand; kankanit_yeerong@cmu.ac.th (K.Y.); jakkapan.s@cmu.ac.th (J.S.); 3 Department of Pharmaceutical Technology, Faculty of Pharmaceutical Sciences, Huachiew Chalermprakiet University, Samutprakarn 10250, Thailand; waranya.ne@gmail.com; 4 Research Center of Pharmaceutical Nanotechnology, Chiang Mai University, Chiang Mai 50200, Thailand; sanuchapreeda@gmail.com; 5 Division of Clinical Microscopy, Department of Medical Technology, Faculty of Associated Medical Sciences, Chiang Mai University, Chiang Mai 50200, Thailand; 6 Department of Entomology and Plant Pathology, Faculty of Agriculture, Chiang Mai University, Chiang Mai 50200, Thailand; chaiwat.t@cmu.ac.th

**Keywords:** *Cordyceps militaris*, cordycepin, nanoemulsion, skin delivery, antioxidant, irritation

## Abstract

This study aimed to develop nanoemulsions for enhancing chemical stability and dermal delivery of *Cordyceps militaris* extracts. *C. militaris* was extracted by maceration and infusion. The extracts were investigated for cordycepin, phenolic, and flavonoid content. The antioxidant activity was investigated by in vitro spectrophotometric methods. The irritation profile was investigated by hen’s egg-chorioallantoic membrane test. Nanoemulsions were developed using high-pressure homogenizer. *C. militaris* extract was incorporated into the nanoemulsion and investigated for safety, release profile, permeation, and skin retention. The results demonstrated that water extract (CW) contained the significantly highest content of cordycepin, phenolics, and flavonoids, which were responsible for antioxidant activity. CW was the most potent antioxidant. CW possessed comparable 2,2′-diphenyl-1-picrylhydrazyl radical scavenging activity and lipid peroxidation inhibition to l-ascorbic acid (96.9 ± 3.1%) and alpha-tocopherol (87.2 ± 1.0%). Consequently, ten mg/mL of CW was incorporated into nanoemulsions composing of sugar squalene, Tween^®^ 85, and deionized water. Nanoemulsion, which had the smallest internal droplet size (157.1 ± 2.6 nm), enhanced the stability of CW, had no cytotoxicity effect and no skin irritation, released the most CW (0.9 ± 0.0% *w*/*w* after 24 h), and delivered the highest CW into the skin layer (33.5 ± 0.7% *w*/*w*). Therefore, nanoemulsion was suggested for enhancing the stability and dermal delivery of CW.

## 1. Introduction

*Cordyceps militaris* is a highly valued fungus, which has been used as traditional medicine in China since ancient times [1]. Nowadays, the fruiting bodies of *C. militaris* have been successfully cultivated on a large-scale and widely used for its health beneficial proprieties all over the world [2]. *C. militaris* is an edible mushroom, which contains high nutritional and therapeutic value [3]. There are various biological active compounds, including cordycepin, cordycepic acid, pentastatin, carotenoids, lectins, cordyceps polysaccharides, proteoglycans, terpenoids, steroids, phenolic compounds, etc. [2,3]. Cordycepin (3′-deoxyadenosine), an adenosine analogue isolated from *C. militaris*, has been reported to possess various pharmacological activities, including antioxidant, immunomodulatory, anti-inflammatory, anticarcinogenic, and antiviral agents [3]. Therefore, it has been extensively studied during the last decade [4,5].

Because of its reported antioxidant activities, *C. militaris* has a potential to be used as an active ingredient in cosmetic/cosmeceutical products [3]. Antioxidants are not only needed to prevent deterioration of the cosmetic products but also exert several beneficial effects on the skin [6]. The antioxidant could terminate chain reaction, remove free radical intermediates, scavenge reactive oxygen species (ROS), and finally prevent the oxidative stress [7]. Oxidative stress induced by ROS leads the damage of lipids, proteins, nucleic acids, and organelles, thus resulting in stress, cellular senescence, and finally skin aging [8]. Therefore, the ROS neutralization has been widely known to maintain good dermal health and prevent chronological or photo-aging [9].

However, cordycepin quickly loses its activity in vivo due to the degradation by adenosine deaminase, which is an enzyme that convert the active adenosine to an inactive inosine [10]. Additionally, external environment also affected the stability of cordycepin. Exposure to UVB light, sun light, and high temperature led to the cordycepin degradation [11]. Furthermore, various pH conditions also affected the stability of cordycepin. Tang et al. (2019) reported that cordycepin was more stable in the neutral and alkaline conditions than in acidic conditions [11].

Although *C. militaris* had a potential to be used as active ingredients in cosmetic/cosmeceutical products due to its potent antioxidant activities, there are some limitations of topical applications since its active components degrade easily. Therefore, the present study aimed to develop nanoemulsion systems for enhancing the stability of cordycepin. Additionally, the safety, release profile, and skin delivery of cordycepin from nanoemulsion were also investigated.

## 2. Materials and Methods

### 2.1. Fungi Materials

*C. militaris* dried powder was obtained from Mushroom Research and Development Center (MRDC, Chiang Mai, Thailand).

### 2.2. Chemical Materials

Cordycepin (purity ≥ 98.0%), 6-hydroxy-2,5,7,8-tetramethylchroman-2-carboxylic acid (Trolox; purity ≥ 95.0%), l-ascorbic acid (purity ≥ 99.0%), sodium chloride (NaCl), sodium dihydrogen phosphate (NaH_2_PO_4_), disodium phosphate (Na_2_HPO_4_), sodium phosphate (Na_3_PO_4_), sodium carbonate (Na_2_CO_3_), sodium thiocyanate (NaSCN), sodium acetate (C_2_H_3_NaO_2_), calcium chloride (CaCl_2_), ferrous chloride (FeCl_2_), ferric chloride (FeCl_3_), ferrous sulfate (FeSO_4_), potassium persulfate (K_2_S_2_O_8_), hydrochloric acid (HCl), linoleic acid, tricine, tris base, 2,2′-diphenyl-1-picrylhydrazyl reagent (DPPH), 2,2′-azino-bis3-ethylbenzothiazoline-6-sulfonic acid (ABTS), 2,4,6-tripyridyl-striazine (TPTZ), Folin-ciocalteu (FC) reagent, bovine serum albumin (BSA), sodium lauryl sulfate (SLS), sugar squalane, Simmondsia chinensis (jojoba) oil, Argania spinosa (argan) oil, polysorbate 20 (Tween^®^ 20), polysorbate 80 (Tween^®^ 80), and polysorbate 85 (Tween^®^ 85) were analytical grade purchased from Sigma-Aldrich (St. Louis, MO, USA). Dulbecco modified eagle medium (DMEM), l-glutamine, amphotericin B, penicillin/streptomycin, and trypan blue were purchased from Invitrogen™ (Grand Island, NY, USA). GlutaMAX™-I supplement was purchased from Thermo Fisher Scientific, Inc. (Grand Island, NY, USA). Ethanol, hexane, ethyl acetate, acetic acid, and dimethyl sulfoxide (DMSO), were analytical grade purchased from Labscan (Dublin, Ireland). HPLC-grade methanol was purchased from Labscan (Dublin, Ireland).

### 2.3. Extraction of C. militaris

#### 2.3.1. Maceration

Briefly, 100 g of dried *C. militaris* powder was macerated in 500 mL of 95% *v/v* ethanol for 24 h at room temperature. The resulting mixture was filtered through Whatman No. 1 filter paper. The *C. militaris* residue was macerated in a fresh solvent, which was 500 mL of 95% *v/v* ethanol. The macerations were performed trice. The filtrate from 3 cycles of macerations were pooled together and the solvent was then removed by rotary evaporator (Buchi Labortechnik GmbH, Essen, Germany). Crude ethanolic extract (CC) was obtained and kept at 4 °C until further used.

#### 2.3.2. Sequential Maceration

Briefly, 100 g of dried *C. militaris* powder was macerated in 500 mL of hexane for 24 h at room temperature. The resulting mixture was filtered through Whatman No. 1 filter paper. The *C. militaris* residue was macerated in a fresh hexane for another two cycles. The filtrate from 3 cycles of macerations in hexane were pooled together and the solvent was then removed by rotary evaporator (Buchi Labortechnik GmbH, Essen, Germany) and a hexane extract (CH) was obtained. Subsequently, the *C. militaris* residue was macerated in ethyl acetate for 24 h at room temperature for 3 cycles. The filtrate from 3 cycles of macerations in hexane were pooled together and the solvent was then removed by rotary evaporator (Buchi Labortechnik GmbH, Essen, Germany) and an ethyl acetate extract (CA) was obtained. Finally, the *C. militaris* residue was macerated in 95% *v/v* ethanol for 24 h at room temperature for 3 cycles. The filtrate from 3 cycles of macerations in 95% *v/v* ethanol were pooled together and the solvent was then removed by rotary evaporator (Buchi Labortechnik GmbH, Essen, Germany) and an ethanolic extract (CE) was obtained. All extracts were kept at 4 °C until further used.

#### 2.3.3. Infusion

Briefly, 100 g of dried *C. militaris* powder was infused in 500 mL of 60 °C DI water for 60 min and then the resulting mixture was left to cool down to the room temperature. The mixture was then filtered through Whatman No. 1 filter paper. The filtrate was frozen to −40 °C and the water was removed by using freeze dryer (FreeZone 4.5 model 7750031, Labconco, Kansas, MO, USA). Water extract (CW) was obtained and kept at 4 °C until further used.

### 2.4. Determination of Chemical Compositions of C. militaris Extracts

#### 2.4.1. Cordycepin Content Determination Using High Performance Liquid Chromatography (HPLC)

HPLC (Hewlett-Packard, Palo Alto, CA, USA) with reversed phase column (Zorbac Eclipse XDB-C18, 4.6 × 150 mm, 0.5 µm) and guard column (Zorbac Eclipse XDB-C18, 4.6 × 50 mm, 0.5 µm) were used to investigate the cordycepin content of the *C. militaris* extract. The sample injection volume was 2 µL. A mixture of 5% methanol and 95% water was used to elute the sample at the flow rate of 1 mL/min and subsequently detected by UV detector set at a wavelength of 260 nm [12]. *C. militaris* solution (1 mg/mL) was filtered through a 0.45 µm nylon filter membrane prior to the injection. Cordycepin was used as a marker for the quantitative determination of *C. militaris* extracts. The standard curve of cordycepin was constructed using the cordycepin concentrations ranging from 2 to 1000 µg/mL. All the experiments were independently performed twice.

#### 2.4.2. Total Phenolic Content Determination by Folin-Ciocalteu (FC) Method

The total phenolic content of the *C. militaris* extract was investigated using the FC method as previously described by Chaiyana et al. (2019), Tachakittirungrod et al. (2007), and Singleton et al. (1999) [13,14,15]. Briefly, FC reagent was diluted in DI water in a ratio of 1:10. Thereafter, 20 µL of each extract (1 mg/mL) was added to 100 mL of FC reagent and incubated for 4 min. A total of 80 µL of 75 g/L sodium carbonate solution was added and incubated at room temperature for 2 h in the dark. The present of phenolic compounds was detected by spectrophotometric measurements using a 96-well microplate reader (BMG Labtech, Ortenberg, Germany) set at the wavelength of 760 nm. The blank sample, which was a mixture of sample solution and the solvent of FC reagent, was performed to reduce an interference from colored samples. Blank of control (DI water) was performed to reduce the interference from each native solvent. Gallic acid was used for the standard curve construction. The results were reported in a term of gallic acid equivalent (GAE), which represented mg GAE/g dry extract. The experiments were independently performed three times.

#### 2.4.3. Total Flavonoid Content Determination by Aluminum Chloride Colorimetric Method

The total flavonoid content of the *C. militaris* extract was determined using aluminum chloride colorimetric method with slight modifications [16,17]. Briefly, 10% aluminum chloride solution and 1 M potassium acetate solution were prepared. Then 100 µL of each sample solution (1 mg/mL) was added to 20 µL of 10% aluminum chloride solution, 20 µL of 1 M potassium acetate solution, and 860 µL of deionized water, respectively. After that, the mixture was incubated at room temperature for 30 min in the dark. The present of flavonoids was detected by spectrophotometric measurements using a 96-well microplate reader (BMG Labtech, Ortenberg, Germany) set at the wavelength of 415 nm. Each sample was analyzed along with its blank, which contains all reagents except 10% aluminum chloride solution. Quercetin was used as a standard compound and the results were reported in terms of mg/g quercetin equivalent (QE). The experiments were independently performed three times.

### 2.5. Antioxidant Activities Determination of C. militaris Extracts

#### 2.5.1. 2,2′-Diphenyl-1-picrylhydrazyl (DPPH) Assay

*C. militaris* extracts and cordycepin were investigated for their radical scavenging activity by using DPPH assay according to the previously described method of Chaiyana et al. (2019) which was slightly modified from Brem et al. (2004) and Blois (1958) [13,18,19]. Briefly, 180 µL of 167 mM DPPH^●^ solution was mixed with 20 µL of the sample solution (1 mg/mL) and incubated for 30 min in the dark. The present of DPPH^●^ was detected by spectrophotometric measurements using a 96-well microplate reader (BMG Labtech, Ortenberg, Germany) set at the wavelength of 520 nm. The blank sample, which was a sample solution in the solvent of DPPH^●^ reagent, was performed to reduce an interference from colored samples. Blank of control, which was the native solvent, was performed to reduce the interference from each solvent. The inhibition against DPPH^●^ was calculated using the following equation;

DPPH inhibition (%) = [1 − (A/B)] × 100(1)

where A is the absorbance difference between sample solution with DPPH^●^ reagent and the sample solution without DPPH^●^ reagent and B is the absorbance difference between DPPH^●^ solution and its native solvent. Ascorbic acid was used as a positive control. The experiments were independently performed three times.

#### 2.5.2. Ferric Reducing Antioxidant Power (FRAP) Assay

*C. militaris* extracts and cordycepin were investigated for their reducing power using the FRAP assay according to the previously described method of Chaiyana et al. (2019), which was slightly modified from Okonogi et al. (2007) and Benzie and Strain (1996) [13,20,21]. Briefly, FRAP solution containing 0.3 M of acetate buffer (pH 3.6), 10 mM of TPTZ solution in 40 mM HCl, and 20 mM of FeCl_3_ in the ratio 10:1:1 was freshly prepared. Then, 20 µL of each sample solution (1 mg/mL) was added to 180 µL of FRAP solution and incubated at room temperature for 5 min. The present of colored Fe^2+^-TPTZ complex was detected by spectrophotometric measurements using a 96-well microplate reader (BMG Labtech, Ortenberg, Germany) set at the wavelength of 595 nm. Ferrous sulfate was used for the standard curve construction. The blank sample, which was a sample solution in the solvent of FRAP reagent, was performed to reduce an interference from colored samples. Blank of control, which was the native solvent, was performed to reduce the interference from each solvent. The results were reported in a term of equivalent concentration (EC_1_), which represented ferric reducing ability equivalent to 1 µM FeSO_4_. Ascorbic acid was used as a positive control. The experiments were independently performed three times.

#### 2.5.3. 2,2′-Azino-bis3-ethylbenzothiazoline-6-sulfonic Acid (ABTS) Assay

*C. militaris* extracts and cordycepin were investigated for their radical scavenging activity by using ABTS assay according to the previously described method of Chaiyana et al. (2019), which was slightly modified from Tachakittirungrod et al. (2007) and Re et al. (1999) [13,14,22]. Briefly, ABTS^•+^ solution was prepared by mixing 7 mM ABTS^•+^ solution and 2.45 mM K_2_S_2_O_8_ solution in a ratio of 2:3 and incubated at room temperature in the dark for 16 h. Then, 20 µL of each sample solution (1 mg/mL) was mixed with 180 µL of ABTS^•+^ solution. The present of ABTS^•+^ was detected by spectrophotometric measurements using a 96-well microplate reader (BMG Labtech, Ortenberg, Germany) set at the wavelength of 750 nm after 5 min incubation. The blank sample, which was a sample solution in the solvent of ABTS^•+^ reagent, was performed to reduce an interference from colored samples. Blank of control, which was the native solvent, was performed to reduce the interference from each solvent. The results were reported in terms of Trolox equivalent antioxidant activity (TEAC). Ascorbic acid was used as a positive control. The experiments were independently performed three times.

#### 2.5.4. Determination of Lipid Peroxidation Inhibition by Ferric Thiocyanate (FTC) Assay

*C. militaris* extracts and cordycepin were investigated for their lipid peroxidation inhibition using FTC according to the previously described method of Poomanee et al. (2015) which was slightly modified from Niehius et al. (1968) [23,24]. Briefly, the mixtures of 50% (*w*/*v*) linoleic acid in DMSO, 1 M sodium thiocyanate (Na_4_SCN), and 3 mM FeCl_2_ were freshly prepared. Then 50 µL of the resulting mixture was mixed with 50 µL of each sample solution (1 mg/mL) and incubated at 37 °C for 60 min. The present of colored ferric thiocyanate complex was detected by spectrophotometric measurements using a 96-well microplate reader (BMG Labtech, Ortenberg, Germany) set at the wavelength of 490 nm. The blank sample, which was a sample solution, was performed to reduce an interference from colored samples. Blank of control, which was the native solvent, was performed to reduce the interference from each solvent. The inhibition of lipid peroxidation was calculated using the following equation;

Lipid peroxidation inhibition (%) = [1 − (A/B)] × 100(2)

where A is the absorbance difference between sample solution with linoleic acid, Na_4_SCN, and FeCl_2_ and the sample solution without these reagents and B is the absorbance difference between linoleic acid, Na_4_SCN, and FeCl_2_ solution and their native solvent. Trolox was used as a positive control. The experiments were independently performed three times.

### 2.6. Irritation Test of C. militaris Extracts by Hen’s Egg Test Chorioallantoic Membrane (HET-CAM) Assay

Examinations for irritation effect for each of the different *C. militaris* extracts were performed using the HET-CAM assay according to Chaiyana et al. (2017) [25]. HET-CAM assay was one of the famous methods used to investigate the irritation effect of natural compounds [26]. In addition, ethical approval was not required since the age of embryo was less than half of incubation period. In the present study, the fertilized hen’s egg was incubated for 7 days at 37 ± 0.5 °C and humidity of 55 ± 7% RH. Prior to the irritation test, the eggshell above air chamber was opened by rotating cutting blade. Then, the inner membrane was carefully removed using forceps to avoid vessel bleeding. After that, 30 µL of (10 mg/mL) sample solutions were exposed to chorioallantoic membrane (CAM). Immediately, the irritation on CAM was observed for 5 min and the time that the irritation signs occurred was recorded. The aqueous solution of SLS (1% *w/v*) and normal saline solution (0.9% *w/v* NaCl) were used as a positive control and a negative control, respectively. Sugar squalene was used as a vehicle control since it was a solvent for all *C. militaris* extracts. The irritation signs, including hemorrhage, vascular lysis, and coagulation on CAM, were observed under the stereomicroscope (Olympus, Tokyo, Japan). The irritation index score (IS) was then calculated using the following equation;

IS = [(301 − t(h))/300 × 5] + [(301 − t(l))/300 × 7] + [(301 − t(c))/300 × 9](3)

where t(h) was the first time vascular hemorrhage occurred, t(l) was the first time vascular lysis occurred, and t(c) was the first time vascular coagulation occurred. Then IS was classified as followed: 0.0–0.9 was no irritation, 1.0–4.9 was slight irritation, 5.0–8.9 was moderate irritation, and 9.0–21.0 was severe irritation [27]. The irritation signs on CAM was observed again after 60 min for the long-term irritation. The experiments were independently performed twice.

### 2.7. Stability of Cordycepin in C. militaris Extracts

#### 2.7.1. Effect of pH

*C. militaris* extracts in various pH conditions, i.e., 5.0, 7.0, and 9.0, were investigated for their physical and chemical stabilities. Each *C. militaris* solution was kept in a light-resistant container at room temperature and the cordycepin content was determined after 1, 2, and 3 months using HPLC analysis as previously described. The experiments were independently performed three times.

#### 2.7.2. Effect of Temperature

*C. militaris* extracts in various temperature, i.e., 4 °C, 45 °C, and room temperature, were investigated for their physical and chemical stabilities. Each *C. militaris* extract was kept dry in a light-resistant container at room temperature and the cordycepin content was determined after 1, 2, and 3 months using HPLC analysis as previously described. The experiments were independently performed three times.

#### 2.7.3. Effect of Light

*C. militaris* extracts in different conditions, i.e., with and without light, were investigated for their physical and chemical stabilities. Each *C. militaris* extracts was kept dry in a transparent and a light-resistant container at room temperature and the cordycepin content was determined after 1, 2, and 3 months using HPLC analysis as previously described. The experiments were independently performed three times.

### 2.8. Development of Nanoemulsion

#### 2.8.1. Nanoemulsion Preparation

Nanoemulsions were prepared by passing the mixture of oil, surfactant, and DI water through a high-pressure homogenizer set at 500 bars for 4 cycles [28,29]. Various factors affecting nanoemulsion development were investigated, including various types and amount of surfactants and oils. To investigate the effect of surfactant type, various surfactans, including Tween^®^ 20, Tween^®^ 80, and Tween^®^ 85 were used at the concentration of 5% *w*/*w* when 10% *w*/*w* sugar squalane was used as an oil phase. To investigate the effect of surfactant amount, various Tween^®^ 85 amounts, including 3, 5, 10, and 15% *w*/*w* were used in the nanoemulsion system when 10% sugar squalane was used as an oil phase. To investigate the effect of oil type, various oils, including jojoba oil, argan oil, and sugar squalane were used at the concentration of 10% *w*/*w* when 5% *w*/*w* of Tween^®^ 85 was used as a surfactant. To investigate the effect of oil amount, various sugar squalane amounts, including 10, 15, and 20% *w*/*w* were used when 5% *w*/*w* of Tween^®^ 85 was used as a surfactant.

#### 2.8.2. Characterizations of Nanoemulsions

Nanoemulsions were characterized for external appearance by organoleptic inspections. Size, polydispersity index (PDI), and zeta potential were investigated by using zeta sizer (Zetasizer^®^, Malvern Instruments Ltd., Malvern, UK). pH was measured using a pH meter.

#### 2.8.3. Stability of Nanoemulsions

The stability of nanoemulsions were investigated after 8 cycles of heating-cooling. In each cycle, the nanoemulsions were stored at 45 °C for 24 h and then at 4 °C for 24 h. Thereafter, the physical appearance, particle size, PDI, and zeta potential were investigated as previously described.

### 2.9. Development of Nanoemulsion Containing C. militaris Extracts

#### 2.9.1. Preparation of Nanoemulsion Containing *C. militaris* Extract

*C. militaris* extract was incorporated into the nanoemulsion systems. The concentration of *C. militaris* extract used in the formulations was related to the concentration, which exerted the antioxidant activities. Briefly, *C. militaris* extract was firstly dissolved in DI water and mixed with the other components. The resulting mixture was then passed through high pressure homogenizer set at 1000 bars for 4 cycles.

#### 2.9.2. Characterizations of Nanoemulsions Containing *C. militaris* Extract

Nanoemulsions containing *C. militaris* extract were characterized for external appearance by organoleptic inspections. Size, polydispersity index (PDI), and zeta potential were investigated by using zeta sizer (Zetasizer^®^, Malvern Instruments Ltd., Malvern, UK). pH was measured by using pH meter.

#### 2.9.3. Stability of Nanoemulsions Containing *C. militaris* Extract

The stability of nanoemulsions containing *C. militaris* extract were investigated after 8 cycles of heating-cooling. In each cycle, the nanoemulsions were stored at 45 °C for 24 h and then at 4 °C for 24 h. After that, the physical appearance, particle size, PDI, and zeta potential were investigated as described above.

### 2.10. Cytotoxicity on Human Keratinocyte (HaCaT) Cells of Nanoemulsions Containing C. militaris Extract by 3-(4,5-dimethylthiazol-2-yl)-2,5-diphenyl Tetrazolium Bromide (MTT) Assay

#### 2.10.1. Human Keratinocyte (HaCaT) Cell Culture and Condition

HaCaT cells (Cell Lines Service, Eppelheim, Germany) were cultured according to a method described by Laothaweerungsawat et al. (2020) in DMEM supplemented with GlutaMAX™-I, 0.25 μg/mL amphotericin B, 10% inactivated FBS, 100 μg/mL streptomycin, 100 U/mL penicillin, and incubated in a humidified atmosphere containing 5% CO_2_ maintained at 37 °C [30].

#### 2.10.2. 3-(4,5-dimethylthiazol-2-yl)-2,5-diphenyl Tetrazolium Bromide (MTT) Assay

The effects of blank nanoemulsions, nanoemulsions containing 10 mg/mL *C. militaris* extract, and 10 mg/mL *C. militaris* extract solution on the viability of HaCaT cell lines were investigated by MTT assay [30]. The final concentration of 10 mg/mL *C. militaris* extract in both solution and nanoemulsion was 100 μg/mL in the MTT system. Trypan blue dye, which could penetrate through the damaged membranes of dead cells, was used for the cell viability measurement [31]. Briefly, 7 × 10^3^ cells were seeded into a well of 96-well plate. After allowing for cell adhesion overnight, various concentrations of the test samples were added and incubated in a humidified atmosphere containing 5% CO_2_ maintained at 37 °C for 24 h. Thereafter, 0.5 mg/mL MTT was added and incubated in the same condition for another 4 h. DMSO was then added to lyse the cells and dissolve the formazan crystals. Subsequently, the present of violet formazan crystals was detected by spectrophotometric measurements using a multimode detector Sp (220–1000 nm) (SPECTROstar Nano, BMG Labtech, Offenburg, Germany), set with the optical density of 570 nm, corrected by the reference wavelength of 690 nm. The cell viability was calculated in respect to the control cells. The experiments were independently performed three times.

### 2.11. In Vivo Irritation Test in Human Using Human Patch Test

The ethic for human research, ethics approval number 04/2563, was approved by the Human Research Ethics Committees, Faculty of Pharmacy, Chiang Mai University. The skin irritation investigation was performed in 30 healthy volunteers by using human patch test with some modifications [32,33]. The selected nanoemulsions containing *C. militaris* extract (NE1) and its blank nanoemulsion were applied on the inner lower arm skin for 4 h. During the study, the skin irritation was observed at 15, 30, 60, 120, 180, and 240 min. After that the patch was taken off and the skin irritation was further assessed at 24, 48, and 72 h.

### 2.12. Release Profile of C. militaris Extract from Nanoemulsions

Nanoemulsions containing *C. militaris* extract were placed in dialysis bags. PBS pH 5.5 was used as an acceptor media. The temperature of the media solution was set at 31 °C. The media solution was then withdrawn in various time duration within 24 h and the cordycepin content was analyzed by HPLC. Each nanoemulsions was investigated along with its blank, which contained no *C. militaris* extract. The release profile of *C. militaris* extract from a solution was also be investigated. The experiments were independently performed three times.

### 2.13. Skin Permeation and Skin Retention of C. militaris Extract from Nanoemulsions

#### 2.13.1. Skin Preparation

The skin of stillborn piglet from the flank area was carefully cut with a surgical blade and washed with PBS pH 5.5. Then the skin was wrapped and kept at −20 °C [34].

#### 2.13.2. Skin Permeation Determination

Skin permeation was investigated by using Franz diffusion cells. Each sample was placed in the donor section. PBS pH 7.4 was used as a receptor media [34], which was withdrawn in various times. Cordycepin content was analyzed by using HPLC. Each nanoemulsion system was investigated along with its blank, which contaiend no *C. militaris* extract. The skin permeation of *C. militaris* extract from the solution was also investigated. The experiments were analyzed in triplicate.

#### 2.13.3. Skin Retention Determination

*C. militaris* extract retained in the skin layer was investigated by using the skin from skin permeation study. Briefly, the skin was rinsed with tap water. The *C. militaris* extract was extracted by using methanol and PBS pH 7.4 in the ratio 1:1 by using a homogenizer [34]. The *C. militaris* extract was analyzed by using HPLC. The experiments were analyzed in triplicate.

### 2.14. Statistical Analysis

Data was analyzed and reported as mean and standard division (S.D.). The statistical analysis was processed by *t*-test and ANOVA using SPSS program (SPSS Statistics 17.0, IBM Corporations, New York, NY, USA). Statistically significant difference was denoted when *p* < 0.05.

## 3. Results and Discussion

### 3.1. C. militaris Extracts

The external appearance and yields of *C. militaris* extracts are shown in Table 1. All *C. militaris* extracts were brownish semisolid mass, except CW, which was brownish powder. The likely explanation of the semisolid appearance of some *C. militaris* extracts might be because some chemical constituents, which were oil or wax-like components, were extracted by nonpolar and semi-polar solvents, i.e., hexane, ethyl acetate, and ethanol. CC yielded the significantly highest extract content, followed by CE, CH, CA, and CW, respectively (*p* < 0.05). The results noted that different extraction methods led to differences in the yield of *C. militaris* extracts. Infusion yielded the lowest extraction amount since it has been known as the extraction method that yield a dilute solution of the readily soluble constituents of plant materials [35]. On the other hand, maceration yielded higher extract content. The likely explanations were due to longer duration of extraction and several cycles of maceration. Besides, sequential extraction gave lower yield after already a few extraction cycles. Therefore, lower yield of CE than CC was due to nonpolar and semi-polar compounds that had already been removed by hexane and ethyl acetate [25].

### 3.2. Chemical Composition of C. militaris Extracts

The content of cordycepin, phenolics, and flavonoids in *C. militaris* extracts were well related among each other, as shown in Table 2. CW contained the significantly highest cordycepin, phenolics, and flavonoids content (*p* < 0.05), followed by CC, CE, CA, and CH, respectively. It was remarked that *C. militaris* extracts, prepared by using higher polar solvents, contained higher content of the bioactive compounds. The explanation was because of a high polarity of cordycepin due to its amine and hydroxyl groups. On the other hand, high polarity of phenolics and flavonoids were because of their hydroxyl group in a phenolic ring. Therefore, cordycepin, phenolics, and flavonoids were polar compounds extracted well using a polar solvent. The results were well in accordance with Quy et al. (2019), who reported that solvent polarity and extraction time affected the cordycepin content of *C. militaris* fruiting body [36]. Besides, Abarca-Vargas et al. (2016) reported that phenolic compounds were more efficiently extracted by high polar solvents and the total phenolic content is obviously related to type of solvent and its polarity index [37]. Similarly, Widyawati et al. (2014) reported that flavonoids were more efficiently extracted by high polar solvents [38].

### 3.3. Antioxidant Activities of C. militaris Extracts

Antioxidant activities of *C. militaris* extracts are show in Table 3. Four different antioxidant assays were used in the present study to confirm the antioxidant potential of *C. militaris* extracts since there are various oxidative mechanisms related to the oxidation process [39]. DPPH, ABTS, and FTC assays were associated with the electron transfer reaction and represented the free radical scavenging activity of the tested compounds [40,41]. In contrast, FRAP assay was associated with the ion reduction process that represented the reducing ability, i.e., an ability of the tested compound to transform ferric ion (Fe^3+^) to ferrous ion (Fe^2+^) [42].

Among different *C. militaris* extracts, CW possessed the significantly highest ferric reducing ability, DPPH^•^ scavenging ability, and lipid peroxidation inhibition (*p* < 0.05). Surprisingly, the radical scavenging activity against DPPH^•^ of CW was comparable to that of ascorbic acid (*p* > 0.05), which is a well-known natural antioxidant compound. Other than reducing ability and lipid peroxidation inhibitory activity of CW, another likely CW antioxidant mechanism was transferring a proton to the DPPH^•^ and scavenge the free radical form of DPPH to its corresponding nitro derivative of DPPH-H [43,44]. Since DPPH^•^ is a free radical that accepts an electron or hydrogen radical to become a stable diamagnetic molecule [45], CW, which possessed the highest DPPH^•^ scavenging ability was suggested to be an antioxidant via the hydrogen donating mechanism. Additionally, CW possessed significantly higher lipid peroxidation inhibition than Trolox (*p* < 0.05), a derivative of alpha-tocopherol which has been reported for a potent antioxidant activity. Therefore, CW would be an attractive antioxidant compound. Both FTC and DPPH assay have been used to evaluate the lipid autoxidation inhibition, when FTC assay measures the antioxidant activity by the inhibition of peroxide production during lipid autoxidation process and DPPH assay measures the free radical scavenging capacity of the antioxidants [46]. Consequently, CW would be a potent antioxidant compound that terminated oxidative chain reactions, including induction, propagation, and termination.

On the other hand, the significantly highest free radical scavenging ability against ABTS^•+^ was detected in CE and CA (*p* < 0.05). Additionally, the ABTS^•+^ scavenging ability of these *C. militaris* extracts were significantly higher than that of ascorbic acid (*p* > 0.05). The likely explanations of potent ABTS^•+^ scavenging ability of CE and CA were due to ABTS^•+^, generated by oxidation of ABTS with potassium persulfate, could be reduced in the presence of hydrogen-donating antioxidants in various solvents including aqueous and lipophilic systems [22]. Since ABTS^•+^ is soluble in both aqueous and organic solvents, it is thus useful in assessing antioxidant activity of samples in different media [47]. Therefore, the antioxidant of CE and CA were more pronounced. Additionally, the results were in a good agreement with the previous study of Ling and Palanisamy (2012), who reported that ethanolic extract of *Arumanis Mangoe* possessed higher ABTS^•+^ scavenging ability than the water extract, and the study of Shalaby and Shanab (2013), who reported that *Spirulina platensis* showed higher antiradical and antioxidant activity (99.55%) with ABTS and absolute methanol extract, while the water extract presented slightly lower activities (95.3%) when tested with DPPH hydrogen reacting radicals [47,48]. However, cordycepin possessed no ABTS^•+^ scavenging ability with the TEAC value about zero (0.0 ± 0.5). The results were well in accordance with Yu et al. (2006), who reported nearly no scavenging effect on ABTS^•+^ of uracil, uridine, adenosine, adenine, and cordycepin [49]. The explanation was due to the solubility limitation of these compounds, especially cordycepin, in TEAC test system [49].

**Figure 1:**
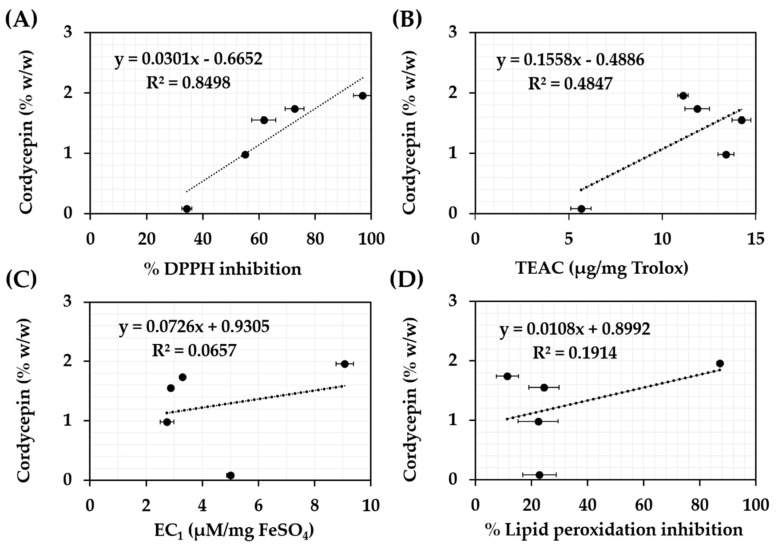
Correlations between cordycepin content and antioxidant activities of *C. militaris* extracts investigated by 2,2′-diphenyl-1-picrylhydrazyl (DPPH) assay (**A**), 2,2′-azino-bis3-ethylbenzothiazoline-6-sulfonic acid (ABTS) assay (**B**), ferric reducing antioxidant power (FRAP) assay (**C**), and ferric thiocyanate (FTC) assay (**D**).

The relations between antioxidant activities of *C. militaris* extracts and their chemical compositions were investigated. The correlations between cordycepin content and antioxidant activities of each *C. militaris* extract are shown in Figure 1. Well and moderate linear correlation was detected in DPPH and ABTS assay with the R^2^ of 0.8498 and 0.4847, respectively. In contrast, no correlation was detected in the others since R^2^ were very low (less than 0.19). Therefore, cordycepin could be noted as a major compound responsible for the radical scavenging activity of *C. militaris* extracts. The ability of cordycepin to scavenge DPPH^•^ was better than ABTS^•+^. The reasons were due to a redox process of DPPH^•^ in regard to homolytical breaking of O–H and N-H bond [44]. Cordycepin, a compound containing amine and hydroxyl groups, were compatible and reacted well with DPPH^•^. The results were in accordance with the previous study that reported that DPPH assay was suggested for determination of antioxidant properties of amines, phenols, or natural compounds [44,50].

On the other hand, chemical components of *C. militaris* extract responsible for high ferric reducing antioxidant power were other compounds than cordycepin because there was almost no relation between the cordycepin content and ferric reducing ability (R^2^ = 0.0657). Therefore, polysaccharides, which were extracted well using DI water, were suggested as noticeable antioxidant components of *C. militaris* extracts. Previous studies remarked polysaccharides, i.e., D-glucose, D-galactose, and D-mannose, as noticeable antioxidant components of *C. militaris* extracts [51,52]. Additionally, polysaccharide fractions containing t-Gal*p* and 1,2-Man*p* have been reported for a potent FRAP value of 78.4 µmol Fe^2+^/g [53]. Similarly, cordycepin was not responsible for lipid peroxidation inhibitory activity of *C. militaris* extract (R^2^ = 0.1914) since hydrophilicity of cordycepin was incompatible with the lipid peroxidation assay [36,54].

**Figure 2:**
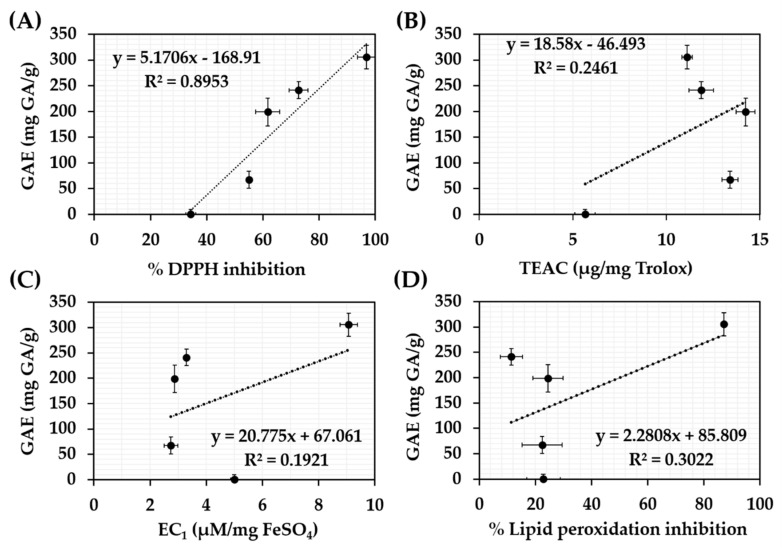
Correlations between total phenolics content and antioxidant activities of *C. militaris* extracts investigated by DPPH assay (**A**), ABTS assay (**B**), FRAP assay (**C**), and FTC assay (**D**).

The correlations between total phenolic content and antioxidant activities of each *C. militaris* extracts are show in Figure 2. Well linear correlation was detected in DPPH assay with the R^2^ of 0.8953. The results were in line with a previous study that reported that DPPH^•^ was a stable free radical, which could be scavenged by a free radical species or an antioxidant, especially purified phenolic compounds [48,55]. However, no correlation was detected in FRAP, ABTS, and FTC assay with the R^2^ less than 0.3. The likely reasons were due to a less pronounced content of phenolic compounds than polysaccharides. A previous study reported that *C. militaris* methanolic extract contained up to 26.72 ± 1.88% of total sugars, whereas, there was only 1.3 × 10^−4^% of phenolic compounds [56]. Therefore, the predominant compounds responsible for the reducing ability, antiradical, and lipid peroxidation inhibitory activity of *C. militaris* extracts could be the polysaccharides.

**Figure 3:**
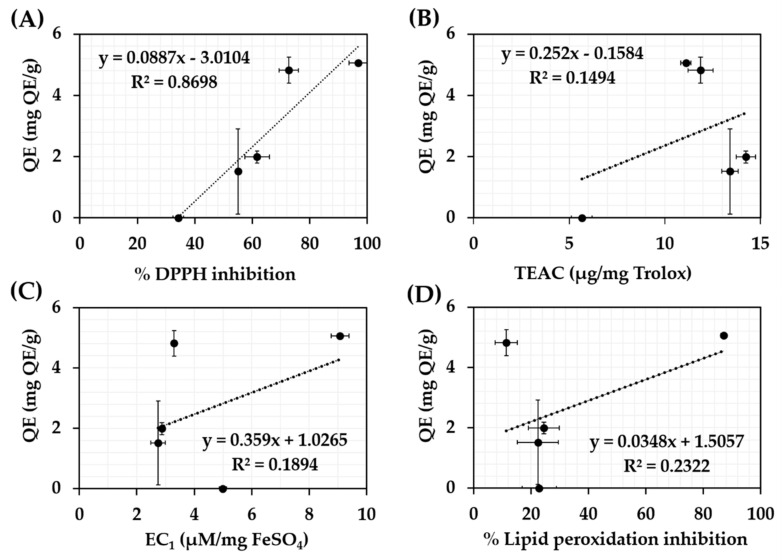
Correlations between total flavonoids content and antioxidant activities of *C. militaris* extracts investigated by DPPH assay (**A**), ABTS assay (**B**), FRAP assay (**C**), and FTC assay (**D**).

The correlations between total flavonoids content and antioxidant activities of each *C. militaris* extracts are show in Figure 3. Similar to the cordycepin and phenolics content, well linear correlation between total flavonoids content and antioxidant activities was detected in DPPH assay with the R^2^ of 0.8698, whereas, no correlation was detected in FRAP, ABTS, and FTC assay. The likely explanations were due to a very little amount of flavonoids in *C. militaris* extracts.

In conclusions, cordycepin, phenolics, and flavonoids were suggested as antioxidants in *C. militaris* extracts. CW was remarked as the most potent antioxidant since it contained the significantly highest content of cordycepin, phenolics, and flavonoids (*p* < 0.05). Besides, CW possessed the significantly highest inhibitory effect against DPPH^•^ radical scavenging and lipid peroxidation with the comparable activities on l-ascorbic acid and alpha-tocopherol, respectively. Therefore, CW was suggested for further development in cosmetic/cosmeceutical products. The suggested effective concentration of CW was 0.1 mg/mL or 0.01% *w*/*w*, since this concentration led to 96.9 ± 3.1% inhibition on DPPH^•^ radical scavenging and 87.2 ± 1.0% on lipid peroxidation. On the other hand, 100 times higher than the effective concentration was suggested for the formulation development since only some active compounds could release and penetrate into the skin layer or reach the target site.

### 3.4. Irritation Profiles of C. militaris Extracts

**Figure 4:**
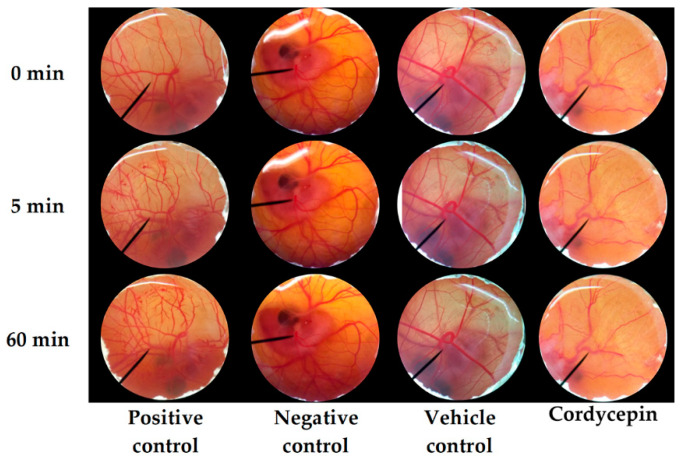
Effect of 1% *w/v* sodium lauryl sulfate (positive control), 0.9% *w/v* normal saline solution (negative control), DI water (vehicle control), and cordycepin on chorioallantoic membrane before (0 min) and after 5 and 60 min of exposure.

The irritation of *C. militaris* extracts was another considerable concern before applying the extract in cosmetic/cosmeceutical products. In the present study, 1% *w/v* SLS, 0.9% *w/v* NSS, and DI water were used as positive, negative, and vehicle control to ensure the accuracy of the HET-CAM test. The results noted that 0.9% *w/v* NSS and DI water induced no irritation sign on the CAM, whereas, 1% *w/v* SLS induced severe irritation. The IS of 1% *w/v* SLS was 14.0 ± 0.2. It was well in accordance with the previous study of Somwongin et al. (2018), who reported that 1% *w/v* SLS induced severe irritation with the IS of 10.6 ± 0.8 [42]. All signs of irritation, including hemorrhage, coagulation, and vascular lysis, were detected in the CAM exposed to 1% *w/v* SLS after 60 min, as shown in Figure 4. On the other hand, both negative and vehicle control induced no irritation signs after 60 min. Therefore, the HET-CAM test was well validated in the present study.

**Figure 5:**
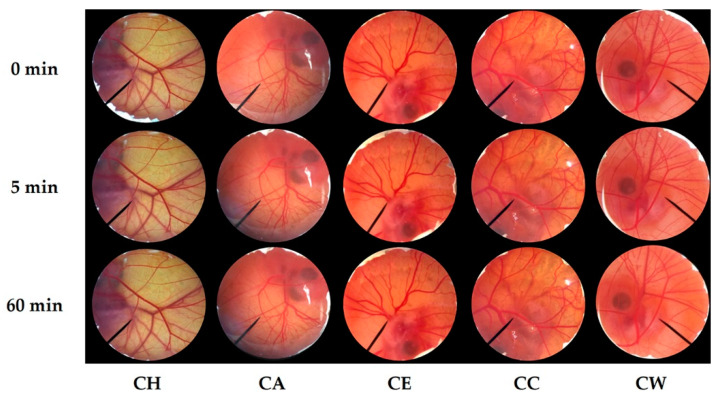
Effect of *C. militaris* extracts, including hexane extract (CH), ethyl acetate (CA), ethanolic extract (CE), crude ethanolic extract (CC), and water extract (CW) on chorioallantoic membrane before (0 min) and after 5 and 60 min of exposure.

According to the suggested concentration of CW in the formulations, 10 mg/mL of *C. militaris* extracts were investigated for the irritation properties. The CAM exposed to *C. militaris* extracts for 5 and 60 min had no irritation signs, as shown in Figure 5**.** Therefore, all *C. militaris* extracts were safe since they induced no irritation. CW was hence suggested as an effective antioxidant and safe for topical applications.

### 3.5. Stability Profile of Cordycepin in C. militaris Extracts

**Figure 6:**
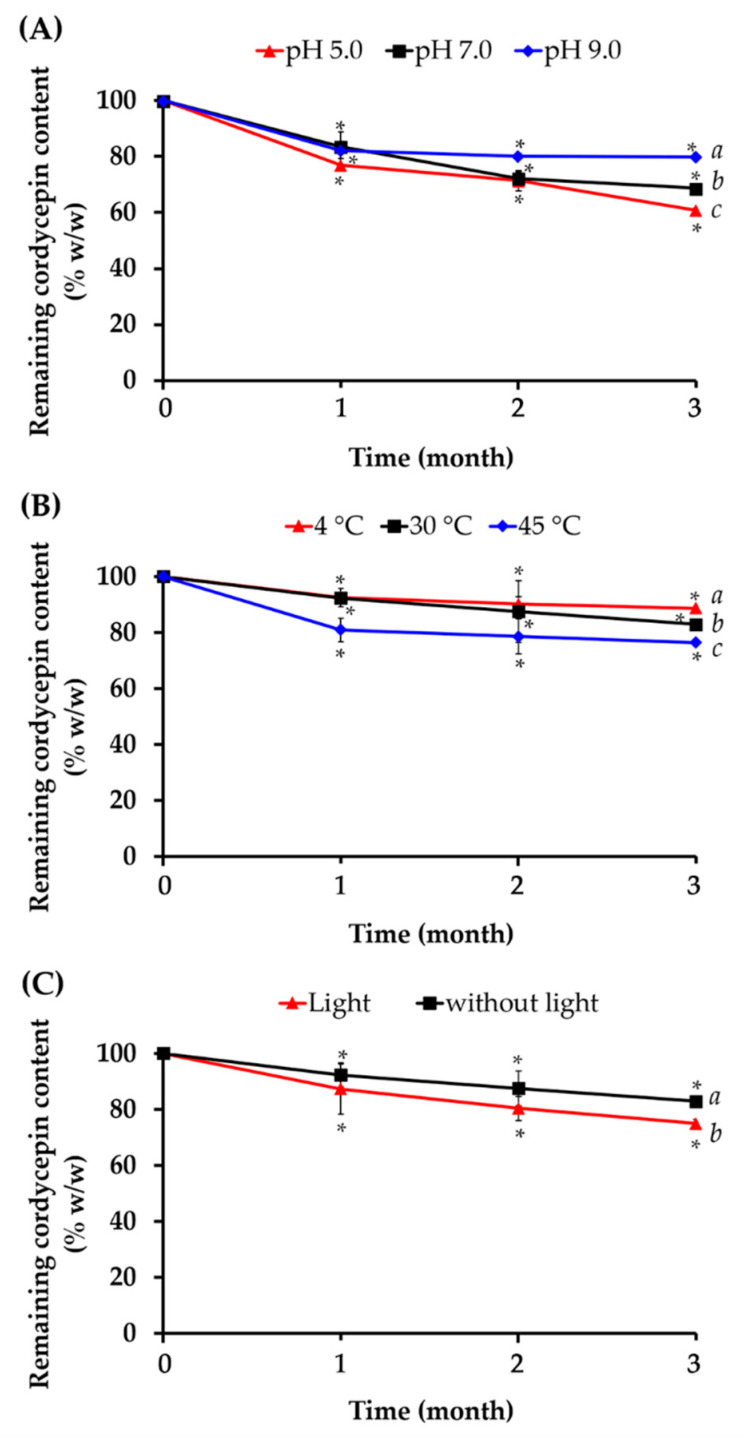
The remaining cordycepin content of *C. militaris* water extract (CW) stored in various pH conditions (**A**), various temperatures (**B**), and dark/light conditions (**C**). Data are show in mean value ± S.D. (*n* = 4). Significant difference of cordycepin content before (day 0) and after 1, 2, and 3 months were analyzed using *t*-test analysis (*p < 0.05).

According to remarkable antioxidant activities of *C. militaris* extracts, especially CW, it has a potential in cosmetic purposes. However, the stability of natural extracts needed to be considerably concerned for applying in cosmetic products. Therefore, various factors affecting the stability of CW have been investigated by means of quantitative determination of cordycepin. The effects of pH on the stability of cordycepin are shown in Figure 6A. Although the physical appearance of each extract was not different from the initial presence, the cordycepin content significantly decreased after 1 month in acidic pH (5.0), neutral pH (7.0), and basic pH (9.0) (*p* < 0.05). Additionally, the remained cordycepin content continuously decreased. Even if cordycepin continuously decreased during longer storage time in all pH conditions, the remaining cordycepin content in basic pH (9.0) was significantly higher than neutral pH (7.0) and acidic pH (5.0) after 3 months, respectively. The results were in a good agreement with previous study from Chutvirasakul (2017), who reported that oxidative decompositions of nucleosides and sugar rings in the cordycepin molecule occurred in acidic conditions [57]. Moreover, temperature and light conditions also affected the remaining cordycepin content of CW. Higher temperature led to lower cordycepin content as shown in Figure 6B, whereas, storage condition with light led to lower cordycepin content as shown in Figure 6C. Therefore, suitable storage condition is very important for CW to maintain the content of bioactive compounds, biological activities, and efficacy. The present study suggested to keep CW in a light-protective container in low temperature, such as in a refrigerator. However, CW storage in basic pH condition was not practical and not suitable for using as a cosmetic active ingredient, since biological pH of the skin was around 5.5. Therefore, further incorporation in nano delivery systems would be a solution to prevent cordycepin degradations.

### 3.6. Nanoemulsion Development

**Figure 7:**
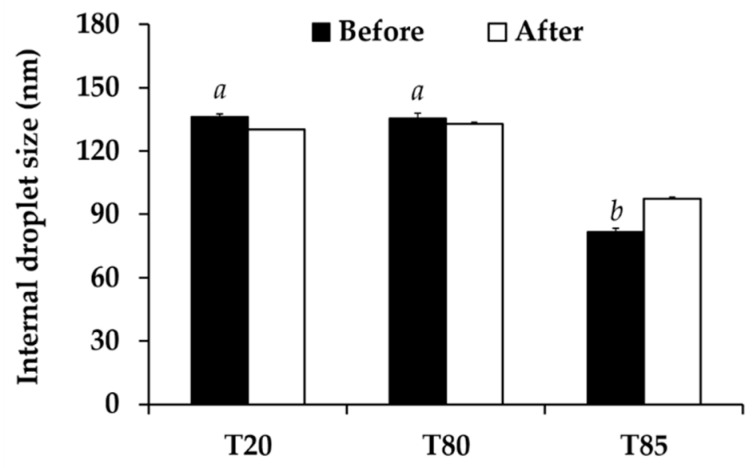
Internal droplet size of nanoemulsions generated using various types of surfactant, including Tween^®^ 20 (T20), Tween^®^ 80 (T80), and Tween^®^ 85 (T85), before and after 8 cycles of heating-cooling condition. The letters a and b denoted significant difference between *nanoemulsions* analyzed using post-hock Tukey ANOVA (*p* < 0.05).

Various factors affecting nanoemulsion development were investigated. The results noted that various types and amount of both oils and surfactants affected the nanoemuslion’s characteristics. Different surfactant types affected the internal droplet size of nanoemulsion systems as shown in Figure 7. Tween^®^ 85 generated the nanoemulsion with the significantly smallest internal droplet size (81.8 ± 1.5 nm) (*p* < 0.05). On the other hand, Tween^®^ 20 and Tween^®^ 80 generated the nanoemulsiond with comparable internal droplet size of 136.1 ± 1.4 and 135.6 ± 2.2 nm, respectively (*p* > 0.05). Besides, all nanoemulsions were stable after 8 cycles of heating-cooling since their external appearance and their internal droplet size were not changed (*p* > 0.05). Therefore, Tween^®^ 85 was selected for further use.

**Figure 8:**
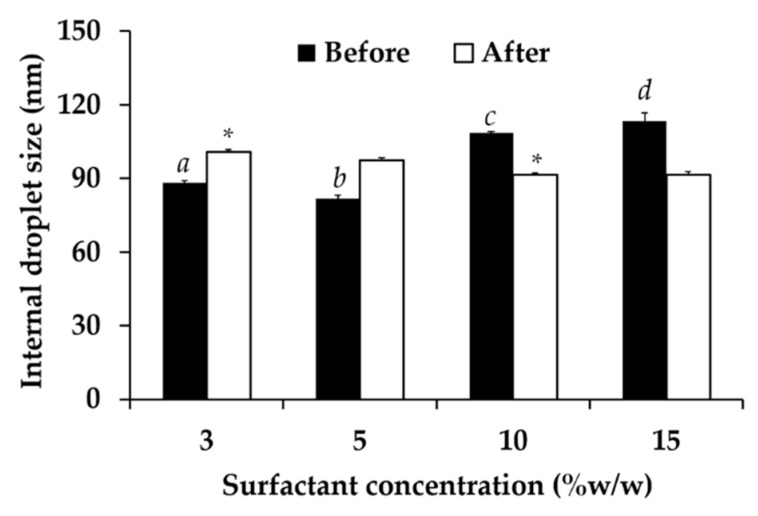
Internal droplet size of nanoemulsion generated using various amount of surfactant, including 3, 5, 10, and 15% *w*/*w*, before and after 8 cycles of heating-cooling condition. The letters a, b, c, and d denoted significant difference between formulations analyzed using post-hock Tukey ANOVA (*p* < 0.05). Asterisk (*) denoted significant difference between before and after the stability test analyzed using *t*-test (*p* < 0.05).

The amount of surfactant also significantly affected the internal droplet size of nanoemulsion systems as shown in Figure 8. The optimal concentration of Tween^®^ 85 was 5% *w*/*w* since it yielded the nanoemulsion with the significantly smallest internal droplet size (81.8 ± 1.5 nm). Lower concentration (3% *w*/*w*) generated the nanoemulsion with larger size since the surfactant was not enough to emulsify the oil phase. Generally, the increasing of surfactant amount allowed the internal droplet to get smaller [58]. However, the concentration of Tween^®^ 85 above 5% *w*/*w* led to larger internal droplet size. The reason was because nanoemulsion transformed into gel-like system when the surfactant amount increased. Higher surfactant amount led the nanoemulsion to have higher viscosity, which led to larger internal droplet size detection. Therefore, 5% *w*/*w* of Tween^®^ 85 was selected for further use.

**Figure 9:**
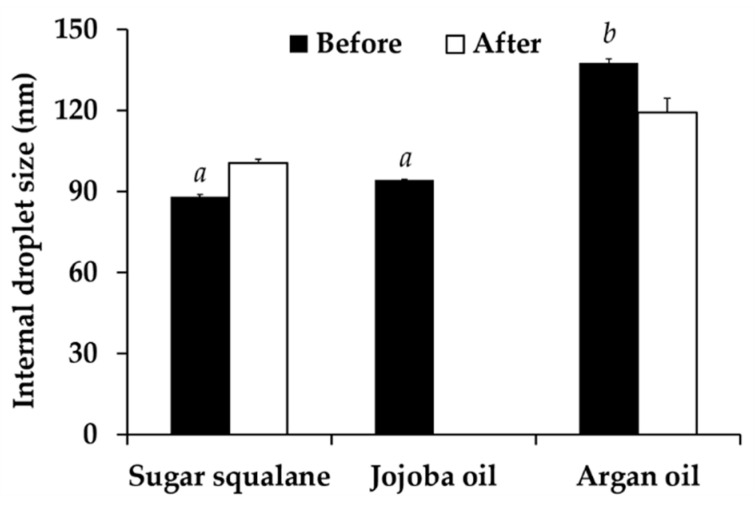
Internal droplet size of nanoemulsion generated using various types of oil, including sugar squalane, jojoba oil, and argan oil, before and after 8 cycles of heating-cooling condition. There letters a and b denoted significant difference between formulations analyzed using post-hock Tukey ANOVA (*p* < 0.05).

Generally, hydrophilic lipophilic balance (HLB) value of surfactant system needed to be related with the required HLB value of an oil phase. The HLB value of Tween^®^ 85 was reported as 11.0 [59]. Therefore, the natural oils having the required HLB closed to 11.0 were used in the present study, including sugar squalane (HLB = 11–12), jojoba oil (HLB = 11.0), and argan oil (HLB = 10–11) [60,61]. The results showed that different oil types had significant effects on the internal droplet size of nanoemulsions. Sugar squalane and jojoba oil yielded the nanoemulsions with the significantly smallest internal droplet size, as shown in Figure 9 (*p* < 0.05). However, the nanoemulsion composing of jojoba oil exhibited the phase separation after 8 cycles of heating-cooling condition, whereas, sugar squalane and argan oil could yield the stable nanoemulsions. Although small internal droplets of nanoemulsions was suggested to be obtained when HLB values of emulsifier system coincided with the required HLB value of an oil phase [61], the present study remarked that different oil types having the same required HLB value generated different nanoemulsion. Therefore, sugar squalene, which generated stable nanoemulsion with the significantly smallest internal droplet size, was selected for further use.

**Figure 10:**
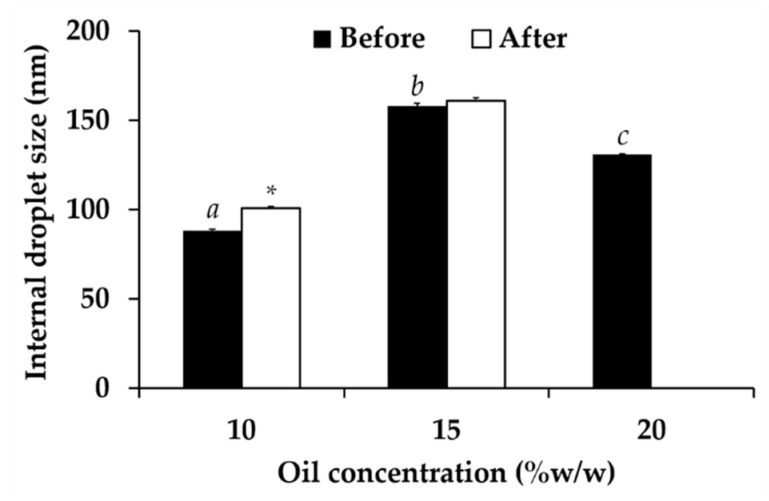
Internal droplet size of nanoemulsion generated using various amount of oil, including 10, 15, and 20% *w*/*w*, before and after 8 cycles of heating-cooling condition. The letters a, b, and c denoted significant difference between formulations analyzed using post-hock Tukey ANOVA (*p* < 0.05). Asterisk (*) denoted significant difference between before and after the stability test analyzed using *t*-test (*p* < 0.05).

The effect of oil amount on internal droplet size of nanoemulsions are shown in Figure 10. Higher amount of oil phase led to larger internal droplet size because the nanoemulsions were O/W type and increasing in the content of internal phase resulted in its larger size. Furthermore, the oil content above 15% *w*/*w* resulted in the phase separation. The likely explanation was due to the large amount of internal oil phase but not enough emulsifier to emulsify the oil, and hence resulting in the instability. The most appropriate oil amount was 10% *w*/*w* since it gave the nanoemulsion with the smallest internal droplet size. Therefore, 10% *w*/*w* of sugar squalane was selected for further investigations.

### 3.7. Nanoemulsion Containing C. militaris Extracts

**Figure 11:**
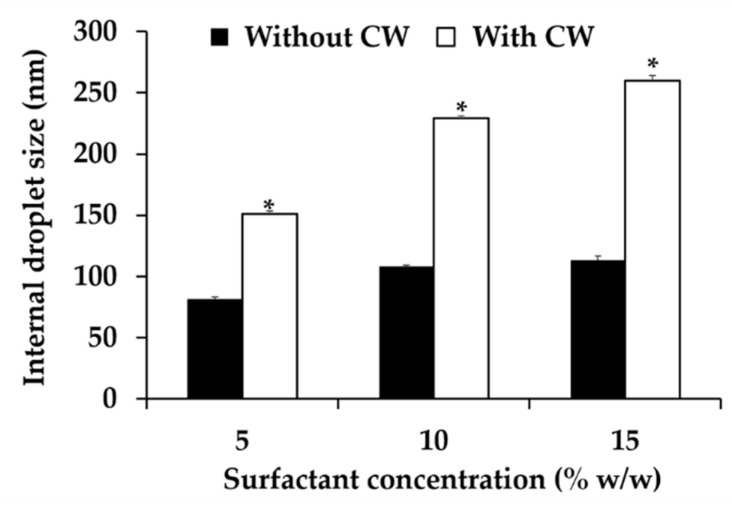
Internal droplet size of nanoemulsions with and without CW. Asterisk (*) denoted significant difference between nanoemulsions with and without CW analyzed using *t*-test (*p* < 0.05).

Three nanoemulsion formulations composing of 10% *w*/*w* sugar squalane and various contents of Tween^®^ 85 (5, 10, 15% *w*/*w*) were selected for CW incorporation. The external appearance of all nanoemulsions containing *C. militaris* extract were homogeneously translucent with milky-orange color. Higher concentration of Tween^®^ 85 led to larger internal droplet size as shown in Figure 11. The internal droplets sized increased from 157.1 ± 2.6 to 229.3 ± 0.1 and 260.1 ± 4.1 nm when the Tween^®^ 85 concentrations increased from 5 to 10 and 15% *w*/*w*, respectively. Additionally, the internal droplet size of all nanoemulsions containing CW extract was larger than their own blank nanoemulsions. Therefore, it could be assumed that CW was entrapped inside the droplet of nanoemulsions.

#### 3.7.1. Physical Stability of Nanoemulsion Containing *C. militaris* Extracts

**Figure 12:**
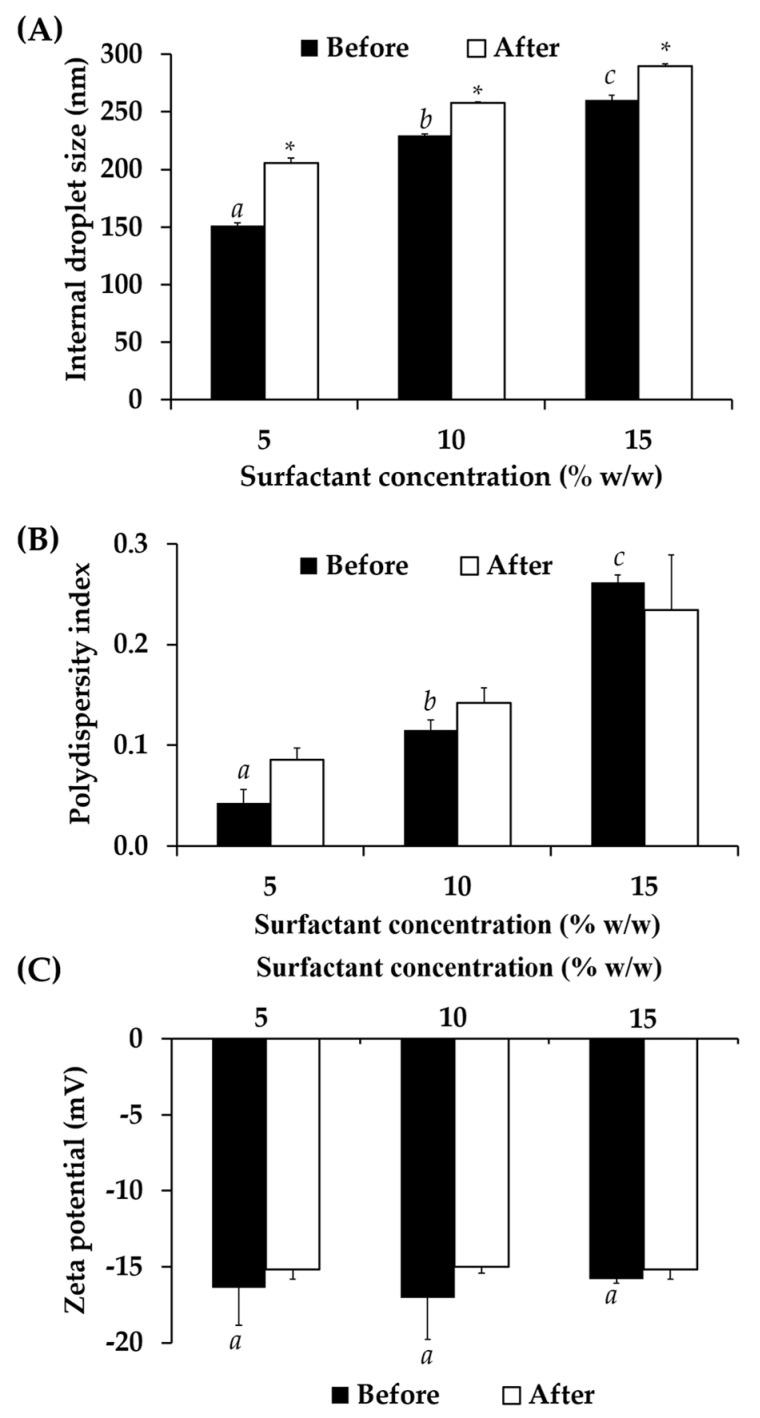
The internal droplet size (**A**), polydispersity index (PDI) (**B**), and zeta potential (**C**) of nanoemulsion containing *C. militaris* extract, before and after 8 cycles of heating-cooling condition. The letters a, b, and c denoted significant difference between formulations analyzed using post-hock Tukey ANOVA (*p* < 0.05). Asterisk (*) denoted significant difference between before and after the stability test analyzed using *t*-test (*p* < 0.05).

All nanoemulsions containing CW had good stability since the external appreance of the formulation remained the same. Additionally, they exhibited pronounced minus zeta potentials, which were ranging from −15.8 ± 0.3 to −17.1 ± 2.7, as shown in Figure 12. Although the internal droplet sizes were significantly increased after the heating-cooling condition, the sizes were in the nano scale, which were less than 300 nm. Additionally, all nanoemulsions exhibited narrow internal droplet size distribution with the PDI less than 0.4.

#### 3.7.2. Chemical Stability of Nanoemulsion Containing *C. militaris* Extracts

**Figure 13:**
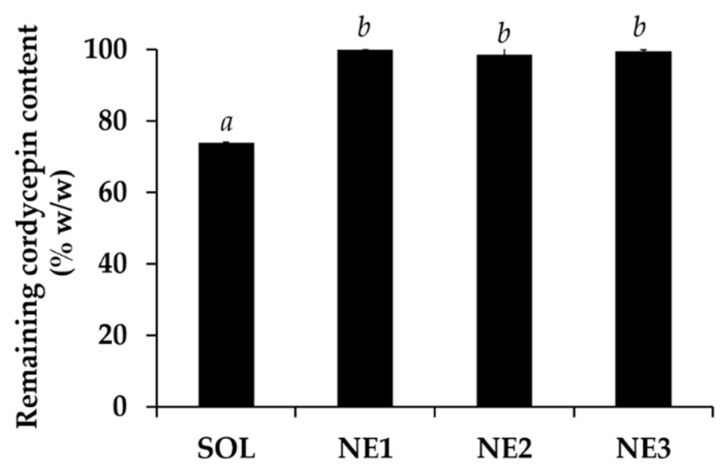
The remaining cordycepin content in CW solution in buffer pH 5.5 (SOL) and nanoemulsions containing various content of Tween^®^ 85, including 5% *w*/*w* (NE1), 10% *w*/*w* (NE2), and 15% *w*/*w* (NE3), after 8 cycles of heating-cooling condition. The letters a and b denoted significant difference between different formulations analyzed using one-way ANOVA with post-hoc Tukey test (*p* < 0.05).

The stability of CW significantly increased after incorporation into nanoemulsions as shown in Figure 13. The remaining cordycepin content significantly increased from 74.0 ± 0.1% *w*/*w* in acidic buffer pH 5.5 to 99.9 ± 0.0, 98.6 ± 1.9, and 99.5 ± 0.4% *w*/*w* in nanoemulsions containing 5, 10, and 15% *w*/*w* of Tween^®^ 85, respectively. The results were in good agreement with a previous study of Sessa et al. (2011), who reported that a nano delivery system could improve the stability and protect the biological active compounds from degradation, especially via the oxidation reaction [62].

### 3.8. Cytotoxicity on HaCaT Cells of Nanoemulsions Containing C. militaris Extract

**Figure 14:**
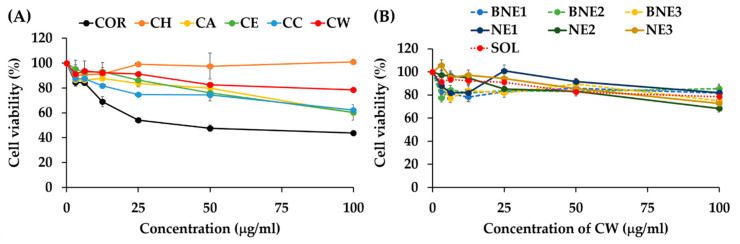
Concentration-viability curve of cordycepin (COR) and *C. militaris* extracts, including hexane extract (CH), ethyl acetate (CA), ethanolic extract (CE), crude ethanolic extract (CC) and water extract (CW) (**A**) and nanoemulsions containing CW (NE1, NE2, and NE3), their blank nanoemulsions (BNE1, BNE2, and BNE3), and CW solution (SOL) on HaCaT keratinocyte cell line (**B**).

The HaCaT cell viability after exposure to *C. militaris* extracts is shown in Figure 14A. It was remarked that cordycepin had some cytotoxicity effects, especially when its concentrations were over 12.5 μg/mL. At this concentration, the HaCaT cell viability was lower than 70%. Additionally, the cell viability decreased up to 50% when the concentration of cordycepin was over 50 μg/mL. On the other hand, all *C. militaris* extracts had lower cytotoxicity effect on HaCaT cells. CW, which possessed the most potent antioxidant activity, had no cytotoxicity effect since the HaCaT cell viability was above 80% at all concentrations tested (3.125–100 μg/mL). Therefore, CW was biocompatible at its suggested effective concentration, which was 100 μg/mL or 0.01% *w*/*w*. CW was hence suggested to be used as natural extract with potent antioxidant activities and safety profile.

The HaCaT cell viability after exposure to blank nanoemulsions and nanoemulsions containing CW is shown in Figure 14B. All nanoemulsions had no cytotoxicity effect. Additionally, the HaCaT cell viability after exposure to the nanoemulsions were not significantly different from those exposed to CW. Therefore, all nanoemulsions were safe and biocompatible. Since HaCaT cells have been widely used as a screening method for the cutaneous irritation prediction [63], it could be concluded that CW and nanoemulsions containing CW were safe, biocompatible, and suggested for further topical applications.

### 3.9. Irritation Effect of Nanoemulsions Containing C. militaris Extract on Human Volunteers

To confirm the safety of nanoemulsions containing *C. militaris* extract for topical applications, both blank nanoemulsions and nanoemulsions containing *C. militaris* extract were tested in 30 healthy human volunteers, aged between 21 to 60 years old (77% of female and 23% of male). The results noted that all nanoemulsions induced no irritation signs on the human skin. Therefore, it could be concluded that nanoemulsions containing *C. militaris* extract were not toxic and biocompatible.

### 3.10. Release Profile of C. militaris Extract from Nanoemulsions

**Figure 15:**
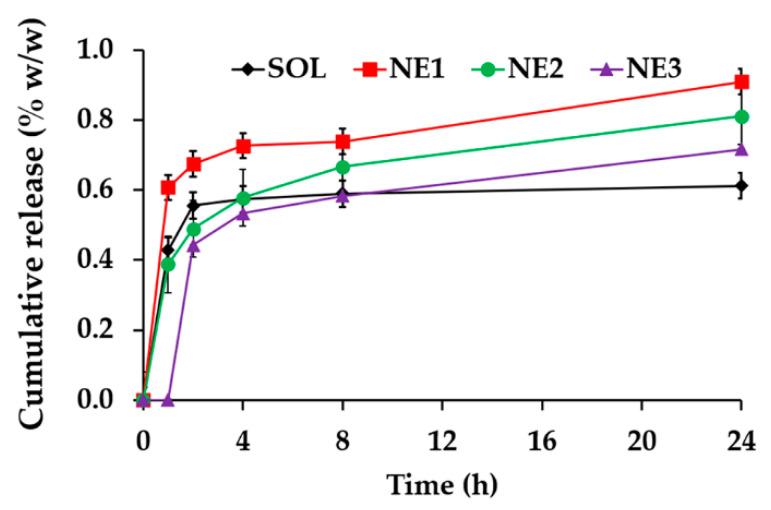
Release profile of *C. militaris* extract (CW) from solution (SOL) and nanoemulsions containing various content of Tween^®^ 85, including 5% *w*/*w* (NE1), 10% *w*/*w* (NE2), and 15% *w*/*w* (NE3). The letters a denoted significant difference between different formulations analyzed using one-way ANOVA with post-hoc Tukey test (*p* < 0.05).

The release profile of CW from nanoemulsions is shown in Figure 15. CW released from all formulations after 1 h, except NE3, which exhibited delay release with 1 h onset. The reason might be due to the largest internal droplet size of NE3 (260.1 ± 4.1 nm) compared to the others, which resulting in the smaller surface area to volume ratio. NE1 demonstrated the significantly highest CW release during 24 h with the cumulative CW release at 24 h of 0.9 ± 0.0% *w*/*w* (*p* < 0.05). The plateau release pattern was observed in the CW aqueous solution after 2 h, whereas, the gradually release pattern after the burst release was observed in all nanoemulsions. The internal droplet size seems to have a significant effect on the CW release since NE1 (157.1 ± 2.6 nm) released more CW than NE2 (229.3 ± 0.1 nm) and NE3 (260.1 ± 4.1), respectively.

### 3.11. Skin Permeation and Skin Retention of C. militaris Extract from Nanoemulsions

Piglet skin has been widely used as an alternative to human skin since there is a close similarity between the modulus of elasticity of young piglet skin and human abdominoplasty and mammaplasty skin [64]. Additionally, a newborn piglet skin model has been widely used for in vitro skin permeation and retention because the lipid composition of pig skin is similar to human skin [65,66] and penetration and retention has been found to be similar for many investigated penetrants [67,68].

**Figure 16:**
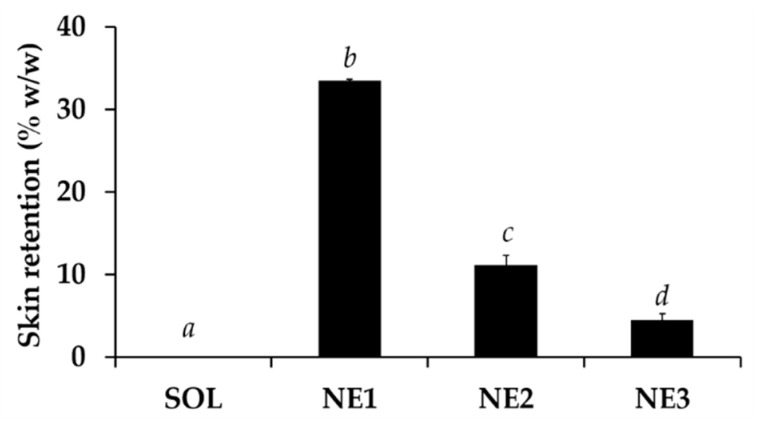
Skin retention of *C. militaris* extract (CW) from solution (SOL) and nanoemulsions containing various content of Tween^®^ 85, including 5% *w*/*w* (NE1), 10% *w*/*w* (NE2), and 15% *w*/*w* (NE3). The letters a, b, c, and d denoted significant difference between different formulations analyzed using one-way ANOVA with post-hoc Tukey test (*p* < 0.05).

CW from all formulations, including solution and nanoemulsions, did not permeate through the skin into the receptor media of Franz cell. These results represented the local effect of CW and could be used to confirm that CW would not induce the systemic effect although it was incorporated in the nano delivery systems. On the other hand, all nanoemulsions enhanced the delivery of CW into the skin layer as shown in Figure 16. NE1, which had the smallest internal droplet size, exhibited the highest skin retention of CW. Additionally, the skin retention content related well with the release ability of the nano formulations. NE1 delivered the significantly highest CW content into the skin layer with the skin retention of 33.5 ± 0.7% *w*/*w*, followed by NE2 (11.2 ± 0.1% *w*/*w*) and NE3 (4.5 ± 1.0% *w*/*w*), respectively. The results were well related with the internal droplet size of each nanoemulsion. Skin retention tends to increase with the decreasing internal droplet size. Therefore, NE1 was suggested as the best nanonoemulsion that could deliver CW into the skin layer

## 4. Conclusions

Among various *C. militaris* extracts, CW contained the significantly highest content of cordycepin, phenolics, and flavonoids, which were responsible for the antioxidant activities of CW (*p* < 0.05). Interestingly, CW possessed comparable radical scavenging activities to that of l-ascorbic acid and comparable lipid peroxidation inhibition to that of alpha-tocopherol. Therefore, CW was remarked as a potent antioxidant for using as active component in cosmetic/cosmeceutical products. The suggested effective concentration of CW was 0.1 mg/mL (0.01% *w*/*w*) since this concentration led to 96.9 ± 3.1% inhibition on DPPH^•^ radical scavenging and 87.2 ± 1.0% on lipid peroxidation. However, ten mg/mL or one percent *w*/*w* of CW was suggested for the cosmetic/cosmeceutical product development since only some CW could release from the formulations and get to the target site. At this concentration, CW was safe for topical applications since it induced no irritation signs on the CAM in HET-CAM test. However, CW was not stable when exposed to light, high temperature, and acidic condition. Therefore, nanoemulsions containing CW were developed using sugar squalene, Tween^®^ 85, and DI water. All nanoemulsions, which were homogeneously translucent with milky-orange color with the internal droplet size ranging from 157.1 ± 2.6 to 260.1 ± 4.1 nm, could enhance the stability of CW. Additionally, NE1, which has the significantly smallest internal droplet size, released the most CW (0.9 ± 0.0% *w*/*w* after 24 h) and delivered the significantly highest CW into the skin layer with skin retention of 33.5 ± 0.7% *w*/*w*. All nanoemulsions were safe, biocompatible, had no cytotoxicity effect on HaCaT cells, and had no irritation effect on human skin. Therefore, NE1 was suggested as the best nanonoemulsion that could enhance the stability of CW and deliver CW into the skin layer.

## Figures and Tables

**Table 1:** External appearance and yields of *C. militaris* extracts.

Extracts	External Appearance	Yield (% *w*/*w*)
CC	Brownish semisolid mass	7.6 ± 2.2 ^a^
CH	Brownish semisolid mass	1.8 ± 0.6 ^c^
CA	Brownish semisolid mass	0.7 ± 0.3 ^d^
CE	Brownish semisolid mass	3.6 ± 1.4 ^b^
CW	Brownish powder	0.5 ± 0.1 ^e^

CC: crude ethanolic extract, CH: hexane extract, CA: ethyl acetate extract, CE: ethanolic extract, CW: water extract. The letter a, b, c, d, and e denoted significant different yield between extracts analyzed using one-way analysis of variance (ANOVA) with post-hoc Tukey test (*p* < 0.05).

**Table 2:** Cordycepin, phenolics, and flavonoids content of *C. militaris* extracts.

Extracts	Cordycepin Content (% *w*/*w*)	Total Phenolics Content (mg GA/g extract)	Total Flavonoids Content (mg QE/g extract)
CC	1.74 ± 0.01 ^b^	241.1 ± 16.6 ^b^	5.06 ± 0.04 ^a^
CH	0.08 ± 0.00 ^e^	0.0 ± 9.8 ^e^	0.00 ± 0.03 ^c^
CA	0.98 ± 0.01 ^d^	67.2 ± 16.7 ^d^	1.52 ± 1.39 ^b^
CE	1.55 ± 0.05 ^c^	199.0 ± 27.1 ^c^	1.99 ± 0.20 ^b^
CW	1.96 ± 0.01 ^a^	305.6 ± 22.8 ^a^	5.06 ± 0.04 ^a^

CC: crude ethanolic extract, CH: hexane extract, CA: ethyl acetate extract, CE: ethanolic extract, CW: water extract, GA: gallic acid, QE: quercetin. The letter a, b, c, d, and e denoted significant different yield between extracts analyzed using one-way ANOVA with post-hoc Tukey test (*p* < 0.05).

**Table 3:** Antioxidant activities of *C. militaris* extracts.

Samples	EC_1_ (µM FeSO_4_/mg)	TEAC (µg Trolox/mg)	Inhibition (%)	
			DPPH	Lipid Peroxidation
l-Ascorbic acid	10.4 ± 0.5 ^a^	12.4 ± 0.0 ^b^	93.8 ± 0.5 ^a^	-
Trolox	-	-	-	65.6 ± 1.7 ^b^
Cordycepin	1.3 ± 0.0 ^e^	0.0 ± 0.5 ^e^	12.6 ± 0.5 ^e^	31.9 ± 3.6 ^c^
CH	5.0 ± 0.1 ^c^	5.7 ± 0.5 ^d^	34.3 ± 1.8 ^d^	22.8 ± 6.0 ^d,e^
CA	2.8 ± 0.3 ^d^	13.4 ± 0.4 ^a,b^	55.0 ± 0.2 ^d^	22.4 ± 7.1 ^d,e^
CE	2.9 ± 0.1 ^d^	14.2 ± 0.5 ^a^	61.7 ± 4.3 ^c^	24.5 ± 5.4 ^d^
CC	3.3 ± 0.0 ^d^	11.9 ± 0.7 ^b,c^	72.7 ± 3.4 ^b^	11.4 ± 3.9 ^e^
CW	9.1 ± 0.3 ^b^	11.1 ± 0.3 ^c^	96.9 ± 3.1 ^a^	87.2 ± 1.0 ^a^

Data were mean ± S.D. (*n* = 3). CH, CA, CE, CC, and CW were *C. militaris* extracts, including hexane extract (CH), ethyl acetate extract (CA), ethanolic extract (CE), crude ethanolic extract (CC), and water extract (CW). The letters a, b, c, d, and e denoted significantly different antioxidant activities among different samples analyzed using Turkey one-way ANOVA (*p* < 0.05).
